# Live-cell imaging reveals single-cell and population-level infection strategies of *Listeria monocytogenes* in macrophages

**DOI:** 10.3389/fimmu.2023.1235675

**Published:** 2023-08-22

**Authors:** Josephine Moran, Liam Feltham, James Bagnall, Marie Goldrick, Elizabeth Lord, Catherine Nettleton, David G. Spiller, Ian Roberts, Pawel Paszek

**Affiliations:** School of Biology, Faculty of Biology, Medicine and Health, University of Manchester, Manchester Academic Health Science Centre, Manchester, United Kingdom

**Keywords:** *Listeria monocytogenes*, macrophage, single cell heterogeneity, phagocytosis, PrfA regulon, listeriolysin

## Abstract

Pathogens have developed intricate strategies to overcome the host’s innate immune responses. In this paper we use live-cell microscopy with a single bacterium resolution to follow in real time interactions between the food-borne pathogen *L. monocytogenes* and host macrophages, a key event controlling the infection *in vivo*. We demonstrate that infection results in heterogeneous outcomes, with only a subset of bacteria able to establish a replicative invasion of macrophages. The fate of individual bacteria in the same host cell was independent from the host cell and non-cooperative, being independent from co-infecting bacteria. A higher multiplicity of infection resulted in a reduced probability of replication of the overall bacterial population. By use of internalisation assays and conditional probabilities to mathematically describe the two-stage invasion process, we demonstrate that the higher MOI compromises the ability of macrophages to phagocytose bacteria. We found that the rate of phagocytosis is mediated *via* the secreted Listeriolysin toxin (LLO), while the probability of replication of intracellular bacteria remained constant. Using strains expressing fluorescent reporters to follow transcription of either the LLO-encoding *hly* or *actA* genes, we show that replicative bacteria exhibited higher PrfA regulon expression in comparison to those bacteria that did not replicate, however elevated PrfA expression *per se* was not sufficient to increase the probability of replication. Overall, this demonstrates a new role for the population-level, but not single cell, PrfA-mediated activity to regulate outcomes of host pathogen interactions.

## Introduction

Specific interactions between pathogenic bacteria and individual host cells decide the course of an infection and its’ outcome. The responses of individual host cells are extremely variable, as exhibited by noisy transcription factor dynamics (1–5) and heterogeneous effector gene production (6–9). In turn, pathogens employ complex strategies to avoid recognition by host cells (10–12), and are able to rapidly adapt to environmental changes to diversify their phenotypes and enhance their survival in the host (13). Consequently, the interactions between host and pathogen at the single cell level are inherently heterogeneous and result in different and “seemingly” probabilistic outcomes (14, 15). For example, only a subset of genetically identical host cells can kill invading *Salmonella* (16), while others allow a pathogen to either persist or replicate to eventually cause a systemic infection (12, 17). Whether different infection outcomes are controlled by the pathogen, the host, or both is not well understood.

The food-borne pathogen *Listeria monocytogenes* is responsible for a number of serious infections with high mortality rates (20-30% in humans) despite antibiotic intervention (18). The potential of *L. monocytogenes* to cause systemic infection depends on the ability to transcytose the intestinal barrier and subvert immune cells to establish infections in the liver and spleen (11, 19). *L. monocytogenes* invades non-phagocytic host cells *via* a membrane-bound vacuole before escaping, replicating in the cytoplasm, and spreading to adjacent cells - all coordinated through the action of the regulatory protein PrfA (20).

The PrfA regulon contains genes required for invasion of non-phagocytic cells (*inlA/B*), phagosome escape (*hly* encoding pore-forming toxin, LLO, together with *plcA* and *plcB*), cytosolic growth (*hpt*) and spread to neighbouring cells through actin polymerisation (*actA*) (21, 22). Regulation of PrfA activity is complex, involving transcriptional and posttranslational control (20, 23–25). It has been shown that the response of *L. monocytogenes* at the single cell level to environmental triggers is heterogeneous, where only a subset of *L. monocytogenes* expressed the PrfA regulated *hly* (26). Likewise, in epithelial cells a small sub-population of pioneer *L. monocytogenes* promoted enhanced cell-to-cell spread (27). *L. monocytogenes* is also capable of switching between different phenotypic states inside the host to diversify its invasion strategies, from an active motile to persistent non-replicative state (28). In turn, genetically identical host cells exhibit different susceptibility to *L. monocytogenes* invasion through the heterogeneity of the endothelial cell adhesions (29). Despite the recent advances highlighting the heterogeneous nature of interactions between bacterial pathogens and host cells, our mechanistic understanding of how the variability in the pathogen and in the host contribute to the overall outcome of infection at the single cell level is limited.

Here we use live-cell confocal microscopy approaches with single bacterial cell resolution to understand interactions between *L. monocytogenes* and host macrophages, a critical event controlling infection (30). We show that infection relies on a fraction of bacteria that can effectively replicate and spread within the macrophage population. Paradoxically, while we found that at the single cell level PrfA regulon expression is heterogeneous and positively correlates with infection outcomes, increased PrfA expression is not sufficient to alter *L. monocytogenes* replication. We also demonstrate that the ability of *L. monocytogenes* to replicate is non-cooperative as multiple bacteria in the same host cell have statistically independent fates, but the overall probability is controlled by the multiplicity of infection (MOI). Furthermore, secreted LLO compromises macrophages’ ability to phagocytose *L. monocytogenes* at higher MOI, while the probability of replication of intracellular bacteria remains constant across different conditions. Overall, these data provide new insights into PrfA-mediated interactions of *L. monocytogenes* and innate immune macrophages and potential new avenues to manipulate infection outcomes.

## Results

### Infection of macrophages results in heterogeneous outcomes at the single cell level

Contrary to established approaches, which typically quantify the “average” behaviour of many bacteria with host cells, we used live-cell confocal microscopy to analyse individual host cell pathogen interactions at a single bacterium resolution. This approach provides insight into the individual infection events that underpin the overall infection process.

**Figure 1:**
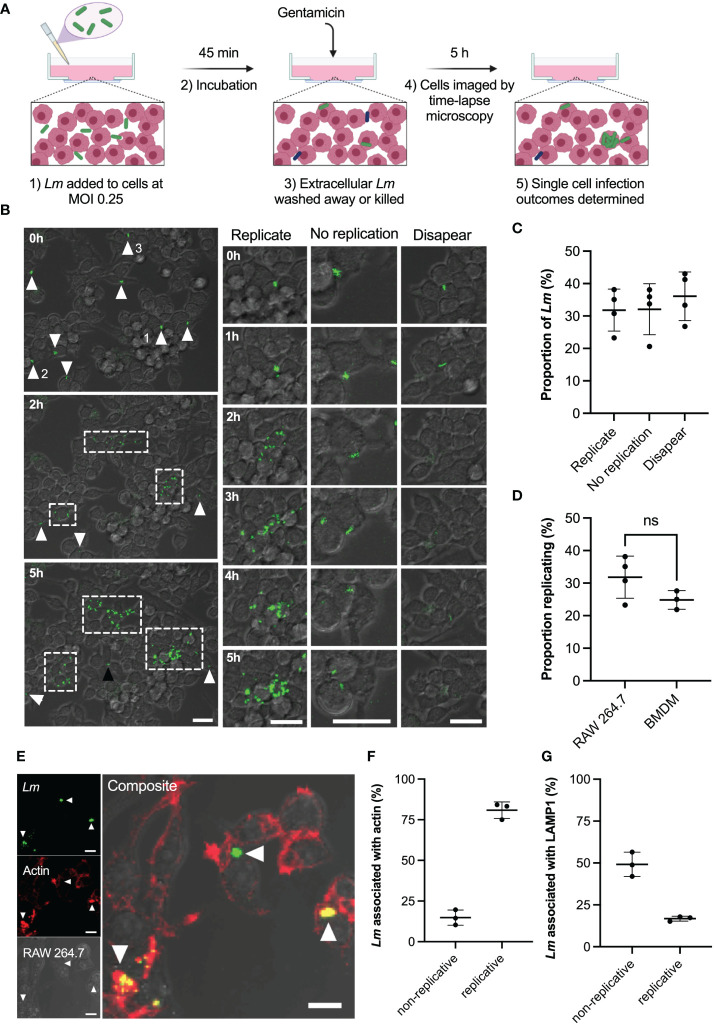
Heterogeneous outcomes of *L. monocytogenes* infection of macrophages at the single cell level. **(A)** Schematic representation of infection protocol: 1) RAW 264.7 macrophages infected with *Lm-*GFP at an MOI of 0.25; 2) Cells and *Lm-*GFP are incubated for 45 mins; 3) Non-adherent *Lm-*GFP are washed away and fresh media containing gentamicin is added to inhibit growth of extracellular bacteria (referred herein as t0); 4) Sample is imaged by time-lapse confocal microscopy for 5 h to determine infection outcomes. **(B)** Representative live-cell microscopy images of RAW 264.7 macrophages (brightfield) infected with *Lm-*GFP (green) from times 0-5 h post gentamicin treatment. White arrows- non-replicative *Lm-*GFP present at t0; white boxes-replicative foci and black arrow: single *Lm-*GFP not visible at t0. The right panels show magnified examples of the 3 outcomes resulting in: (1) replicative infection; (2) non-replicative infection and (3) disappearance. Scale 20μM. **(C)** Proportion of different single cell infection outcomes as depicted in b evaluated at 5 h as a function of the total *Lm-*GFP interactions at t0. Data from four replicates (from 358 individual interactions) shown in circles with mean and SD as solid lines. **(D)** Proportion of replicative invasions for primary BMDMs infected with *Lm*-GFP, evaluated at 5 h as a function of the total *Lm-*GFP associated with BMDMs at t0 (in comparison to data from c). Triplicate data (from 153 individual interactions) shown in circles with mean and SD as solid lines. Statistical significance (ns = non-significant) assessed using Mann-Whitney rank test. **(E)** Representative image of actin staining showing: *Lm-*GFP replicating (arrow pointing down), non-replicating without acting association (arrow pointing left) and non-replicating associated with actin (arrow pointing up). RAW 264.7 macrophages (brightfield) infected with *Lm*-GFP were fixed at 5 h, permeabilised then stained with anti-*Lm* (green) and phalloidin-594 (red). Scale bar 10 μM. Images representative of three replicated experiments. **(F)** Proportions of replicative and non-replicative *Lm*-GFP at 5 h with or without actin staining colocalization as depicted in e (as a function of the total bacteria). Triplicate data shown in circles with mean and SD as solid lines. **(G)** Proportions of replicative and non-replicative *Lm*-GFP at 5 h with or without LAMP1 staining colocalization as depicted in **Figure S1** (as a function of the total bacteria). Triplicate data shown in circles with mean and SD as solid lines.

Monolayers of a murine macrophage cell line (RAW 264.7) and primary murine bone marrow derived macrophages (BMDMs) were infected with *L. monocytogenes* expressing green fluorescent protein (referred herein as *Lm*-GFP) from a chromosomally integrated plasmid (see Materials and Methods). We used a membrane impermeant gentamicin protection assay (where start of the imaging experiment in referred as t0) and a low MOI of 0.25 equivalent to 4:1 host cell to pathogen ratio to exclude multiple invasion events per host cell and thus spatially separate individual host-pathogen interactions (**Figure 1A**). In a typical experiment this resulted in 4.1% of host cells (90 per 2200 cells) harbouring exactly 1 bacterium at t0. An additional 4 host cells were infected with multiple bacteria on average per replicate, an event too rare to account statistically thus removed from subsequent analyses.

Upon infection (**Figure 1B**; **Video 1**) we identified three main outcomes at 5 h post infection resulting in: (1) intracellular replication of bacteria; (2) non-replicative invasion and (3) disappearance. We found that upon invasion of RAW 264.7 macrophages, on average only 32% (±6% standard deviation, SD) of individual host-pathogen interactions resulted in a replicative infection (**Figure 1C**). These replication events were typically initiated within the 2 h post infection and resulted in rapid growth (up to 30 bacteria in 5 h) and spread to neighbouring host cells forming characteristic replicative foci (as indicated by white boxes in **Figure 1B**). Likewise, 32% (±8%) of single-cell interactions resulted in non-replicative invasion, where individual bacteria remained associated with the original host cell for the duration of the experiment. In some cases, bacteria established a new interaction event at a later time point (see black arrow in **Figure 1B**), these bacteria were not included in the analysis. In addition, 36% (±8%) of bacteria disappeared within 5 h post infection from the imaging region. This disappearance is consistent with phagosome killing of bacteria; however, we cannot exclude the possibility that some bacteria escaped to the media. Importantly, the invasion of murine BMDMs at MOI=0.25 resulted in similar infection outcomes; 25% (±3%) of interactions resulted in replicative invasion, which was not statistically different from the macrophage cell line (**Figure 1D**). Overall, these data demonstrate the heterogeneous nature of interactions between *L. monocytogenes* and host macrophages, with only a fraction of bacteria being able to replicate and spread within the host cell population.

The intracellular life cycle of *L. monocytogenes* is well characterised, the bacteria must escape the phagosome into the cytoplasm, where they replicate and accumulate actin for intracellular propulsion (20). We therefore wanted to determine the localisation of replicative vs non-replicative bacteria, to determine if non-replicative bacteria were merely those that failed to escape the phagosome. We used phalloidin staining to detect actin (**Figure 1E**) and lysosomal-associated membrane protein 1 (LAMP1) staining (**Figure S1**) to determine cytoplasmic or phagosomal location, respectively (31–36). We found that 81% (±5%) of replicative bacteria co-localised with actin and thus were present in the host cell cytoplasm (**Figure 1F**), while 17% (±1%) did not colocalise with actin so were predicted to be localised in phagosomes (**Figure 1G**). This is in strong agreement with a previous study using RAW 264.7 cells (36), demonstrating that almost all bacteria present at replicative foci exhibit cytoplasmic localisation. For non-replicative bacteria, we found that 15% (±6%) also showed robust co-localisation with actin staining (**Figure 1F**). This shows that some bacteria are present in the host cytoplasm, but do not replicate. The presence of actin also indicates these bacteria were not destined for autophagy (37). The 49% (±6%) of non-replicative bacteria that exhibited LAMP1 staining (**Figure 1G**) corresponded to intracellular bacteria that failed to escape the phagosome. The remaining 35% of non-replicative bacteria likely represent cells that are not internalised or are cytoplasmic but do not express ActA at the time of measurement, as previously shown (28).

Our live single-cell imaging approach was therefore able to track the heterogeneous outcomes of host and pathogen interactions, with only a third of *L. monocytogenes* seeding subsequent infections. Our localisation assays demonstrate that the majority of replicative *L. monocytogenes* are cytoplasmic, consistent with the ability to escape the phagosome being important for replicative success. However, not all non-replicative *L. monocytogenes* are within phagosomes, indicating phagosomal escape is not the only determinant of replication.

### Elevated PrfA activity does not affect replication at the single cell level

Given that the PrfA regulon plays such a critical role in *L. monocytogenes* virulence, and particularly phagosomal escape (20, 23–25), we wanted to understand the relationship between PrfA activity and replicative outcome at the single cell level. We therefore used reporter strains for the PrfA regulated *hly* and *actA* genes within our live single cell assay to investigate correlations between PrfA activity and replicative outcomes.

To follow virulence gene expression in individual cells we developed dual reporter *Lm* strains, in which promoter (*Phly* or *PactA*) reporter gene fusions were chromosomally integrated to drive GFP expression, in addition to constitutively expressed tagRFP (see Materials and Methods). In agreement with previous analyses (26), we found that *Phly* mediated expression of GFP (*Phly* -GFP) exhibited substantial heterogeneity in *L. monocytogenes* when cultured in tissue culture cell media for up to 1.5h, with only a subset of cells reaching high expression levels (**Figure S2**). While a control *ΔprfA* mutant showed no detectable *Phly*-GFP expression, the PrfA* strain, in which PrfA is constitutively activated (24), exhibited substantially elevated fluorescence levels and reduced cell-to-cell variability compared to that of the wild type (coefficient of variation 0.93 vs. 0.62, **Figure S2**).

**Figure 2:**
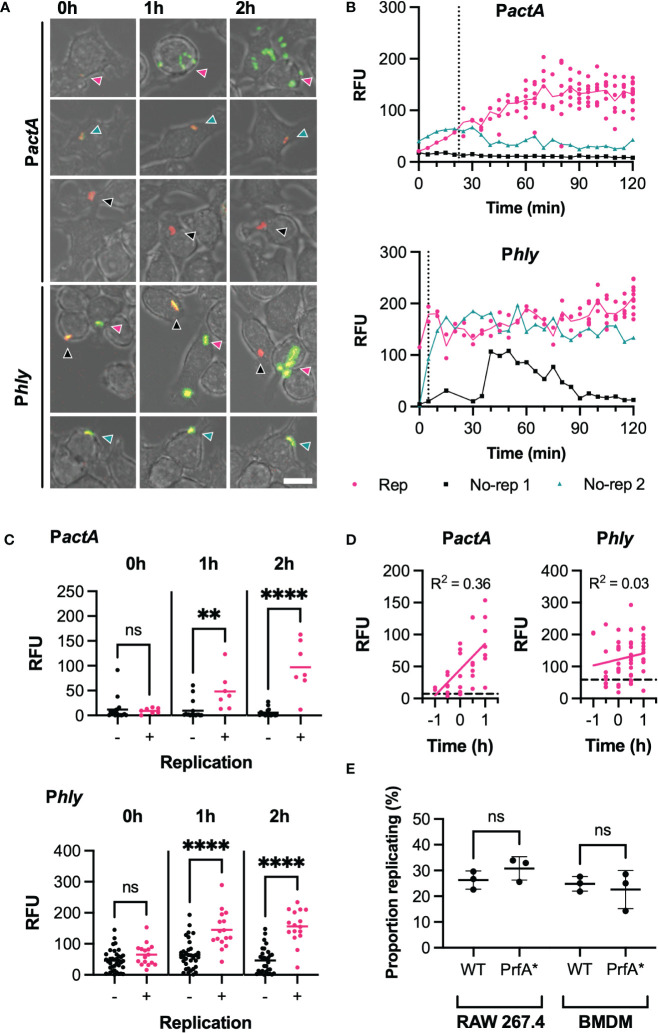
PrfA activity correlates with, but does not determine, infection outcome **(A)** Representative images of live-cell *Lm*-dsRed P*actA*-GFP (P*actA*) or *Lm*-dsRed P*hly*-GFP (P*hly*) infection of RAW 264.7 macrophages at MOI 0.25. Shown are RAW 264.7 macrophages (brightfield) infected with *Lm*-dsRed P*actA*-GFP or *Lm*-dsRed P*hly*-GFP (red) and expressing GFP under the control of *actA* or *hly* promoter region (green) at 0h, 1h or 2h. Arrows indicate the individual *L. monocytogenes* or replicative foci, for replicative (pink), non-replicative 1 (black) or non-replicative 2 (teal). Scale bar 10 μM. **(B)** Reporter expression trajectories over time for representative individual *Lm*-dsRed P*actA*-GFP (P*actA*) or *Lm*-dsRed P*hly*-GFP (P*hly*) and their daughter cells during infection of RAW 264.7 macrophages at MOI 0.25 from 0-2h. Individual tracked bacteria that were replicative (Rep, pink) or non-replicative (no-rep 1, black; no-rep 2, teal) indicated by circles and correspond to the images in **(A)** GFP intensities measured as relative fluorescence units (RFU) every 5 min, for up to 12 (P*actA*) or 10 (P*hly*) replicative daughter cells. Mean RFU (solid lines) and time of first replication (dotted line) also shown. **(C)** Reporter fluorescence expression for *Lm*-dsRed P*actA*-GFP (P*actA*) or *Lm*-dsRed P*hly*-GFP (P*hly*) cells during infection of RAW 264.7 macrophages at MOI 0.25. Data from 3 replicate experiments for individual non-replicative bacteria (black circles, total 37 for P*hly*, 17 for P*actA*) or representative individual bacteria from all replicative foci (pink circles, total 16 for P*hly*, 7 for P*actA*) and their mean (solid lines) shown for 0, 1 and 2h. GFP intensities measured as relative fluorescence units (RFU). Statistical significance (ns = non-significant, ** = p-value <0.01, **** = p-value <0.0001) assessed using Mann-Whitney rank test. **(D)** Reporter fluorescence expression by time before/after first replication for *Lm*-dsRed P*actA*-GFP (P*actA*) or *Lm*-dsRed P*hly*-GFP (P*hly*) during infection of RAW 264.7 macrophages at MOI 0.25. Data from 3 replicates for representative individual bacteria from all replicative foci (pink circles), simple linear regression for replicative data (pink solid line) and average non-replicative expression (black broken line) shown (with corresponding correlation coefficient R^2^). GFP intensities measured as relative fluorescence units (RFU). **(E)** Proportion of WT or PrfA* *Lm*-GFP replicating upon infection of RAW 264.7 or BMDM primary macrophages at MOI 0.25. Individual data from three experiments (circles) with mean and SD (solid lines). Statistical significance assessed with Mann-Whitney rank test.

We hypothesised that the ability to establish a replicative invasion was dependent on the level of PrfA activity in individual bacteria. We therefore used confocal microscopy to follow temporal regulation of PrfA activity *via Phly-*GFP and *PactA-*GFP expression and the fate of individual bacteria upon infection of RAW 264.7 macrophages (**Figure 2A**; **Videos 2**, **3**). First, we found that following invasion, representative replicating bacteria tracked with a high temporal resolution (every 5 min) exhibited induction of PrfA activity (**Figure 2B**, pink arrowheads), consistent with PrfA induction which is known to occur when *L. monocytogenes* is inside a host cell (38). Specifically, *Phly*-GFP expression rapidly increased and was maintained within the 2 h time window, notably through multiple division events. Similarly, *PactA*-GFP was robustly induced with delayed kinetics, as previously indicated at the population level (39) and predicted by differences in PrfA-PrfA box binding specificities between *hly* and *actA* promoter regions (40). The robust activation from both promoters was observed through multiple divisions, which suggests ongoing transcription. In contrast, representative bacteria that did not replicate showed lower PrfA activity, however at least one non-replicative tracked bacterium robustly upregulated *Phly*-GFP expression (depicted in blue in **Figures 2A, B**, black arrowheads).

To analyse the patterns of PrfA activity more systematically, we quantified *Phly*-GFP and *PactA*-GFP at selected times post invasion. At t0, the levels of *Phly*-GFP and *PactA*-GFP expression were not statistically different between those bacteria that went on to establish a replicative infection and those that did not. However, at 1 and 2 h post invasion, replicative bacteria induced significantly higher *Phly*-GFP or *PactA*-GFP reporter expression, compared to those bacteria that did not replicate (**Figure 2C**). While some non-replicative bacteria exhibited substantial *Phly-*GFP expression over time, corresponding *PactA*-GFP fluorescence always remained at low basal levels. This suggested a correlation between the elevated levels and activity of PrfA required for activation of the *PactA* promoter and the infection outcome. To refine these analyses, we estimated the time to the (first) replication from the time-lapse imaging data, which highlighted a large variability in onset of replication (0 to 90 mins from t0) with some bacteria able to replicate immediately following gentamicin treatment at t0 (**Figure S3**). Normalisation of temporal PrfA trajectories according to replication time (**Figure 2D**) demonstrated that temporal increases of *PactA*-expression was a very strong indicator for the replicative invasion, highlighted by a high temporal correlation of expression levels for *PactA* (R^2^ = 0.36, p-value <0.001) but not *Phly* (R^2^ = 0.03, p-value= 0.21).

To test this apparent correlation functionally, and to specifically quantify if enhanced PrfA activation increases replication probability, we analysed infections with the PrfA*-strain, in which PrfA shows higher activity compared to a WT strain (**Figure S2**). We found no statistical difference in the replication probability of wild type or PrfA* strains in RAW 264.7 and BMDMs macrophages (at MOI 0.25), demonstrating that increased PrfA activity *per se* was not sufficient to induce replication.

### Invasion of individual bacteria in the same host cell is non-cooperative

**Figure 3:**
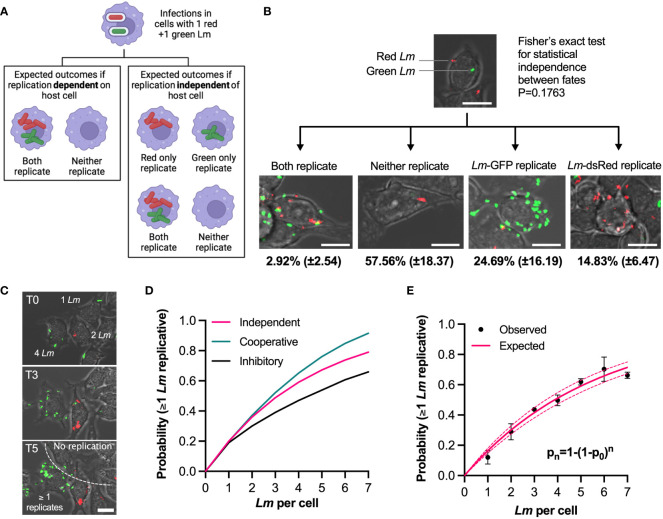
Individual bacteria exhibit independent fates in the same host cell **(A)** Experimental rationale: fates of red and green *L. monocytogenes* in the same host cell determine pathogen and host contribution to replicative invasion. If both bacteria share the same fate in the same host cell, the host environment controls the outcome, if fates are independent, bacteria control the outcome. **(B)** Representative images of different outcomes of RAW 264.7 macrophages simultaneously infected with *Lm*-dsRed and *Lm*-GFP at combined MOI 5 (at 1:1 ratio). Shown are the mean proportion and SDs of different infection outcomes of triplicate data subset of cells with one *Lm-*dsRed and one *Lm*-GFP at t0 (total 167 cells). Scale bar 10 μM. **(C)** Quantification of the replication probability for multiple invasion events per host cell. Representative images of data from b, with cells harbouring 1-4 bacteria at t0. Increased pathogen number over time (as highlighted on the image) indicates that at least one bacterium replicated. Scale bar 10 μM. **(D)** Schematic representation of collective invasion strategies: (1) Cooperative invasion: multiple bacteria in the same cell promote each other’s replication, leading to increased replication probability; (2) non-cooperative invasion: bacterial replication is independent in the same host (probability of replication given by statistical independence p_n_=1-(1-p_0_)^n^, where n is the number of bacteria, p0 replication probability for 1 bacteria); (3) inhibitory invasion: reduced bacterial replication probability as number of bacteria increases due to enhanced immune response. **(E)** Probability that at least one *L. monocytogenes* replicates as a function of number of bacteria per host cell at t0. Shown in black are observed probabilities (mean and SDs, based on three replicate experiments). Solid pink line depicts expected probabilities assuming statistical independence, and 95% confidence intervals in broken lines.

As our data indicated that PrfA activation does not determine intracellular replication, we next wanted to address the fundamental question of whether *L. monocytogenes* ability to replicate is determined by the host or the pathogen, or both. To discriminate between these possibilities, we simultaneously infected macrophages with *L. monocytogenes* expressing either green (*Lm*-GFP) or red (*Lm*-dsRed) fluorescent protein using a combined MOI of 5 (at 1:1 ratio between red and green bacteria). This enabled us to determine outcomes of multiple invasion events per individual host cell. For example, if two bacteria share the same fate upon invasion of the same host cell (e.g., the ability to replicate) it would suggest that the single cell outcome is controlled by the host environment, with some host cells being more permissive to replication than others. Conversely, if fates are statistically independent, the infection outcome is controlled by the bacteria (**Figure 3A**).

To test these possibilities, we first focused on a subset of host cells that at t0 were infected with exactly one green and one red bacteria (167 cells from triplicate experiments, **Figure 3B**; **Video 4**). We found that the marginal probabilities that green or red bacteria replicate, although slightly different from each other, p_G_= 0.257 (±0.16) vs. p_R_=0.148 (±0.07), respectively, were not statistically different (Mann-Whitney test p value 0.4). Assuming the statistical independence, the expected probability that both red and green bacteria replicate in the same host cell is the product of their marginal probabilities, p= p_G_ x p_R_ = 0.257 x 0.148 = 0.036 (±0.04), while the expected probability that neither red nor green replicate is p_n_=(1-p_G_) x (1-p_R_)= 0.63 (±0.19). The data show the probability that both green and red bacteria replicated to be 0.029 (±0.025), while the probability that neither replicated was 0.58 (±0.18). These could not be statistically distinguished from the expected probabilities (Fisher exact test p value 0.18). Therefore, the fate of individual bacteria in the same host is independent from each other, suggesting it is the behaviour of individual *L. monocytogenes* cells that determine the overall outcome of the infection.

The presence of multiple bacteria per host cell raises questions about replication strategy of *L. monocytogenes*; do multiple bacteria cooperate to increase the likelihood of replication or in contrast, are multiple bacteria cleared more efficiently by host cells. To address this, we tested whether the probability of replication depended on the number of *L. monocytogenes* associated with cells at t0, based on the live-cell microscopy movies that accurately demonstrate whether the number of bacteria per host cell increases over time or not (**Figure 3C**). We define expected probability that at least one bacterium replicates using the formula p_n_=1-(1-p_0_)^n^ where *n* denotes the number of bacteria per cell at t0 and *p_0_* is the replication probability for one bacterium per host cell. If there is cooperatively between individual bacteria in the host cell enabling more efficient replication then you would predict probabilities greater than p_n_. In turn, lower probabilities would indicate that host cell is sensing multiple bacteria and actively limiting their spread (**Figure 3D**). We found that the observed probabilities exhibited sub-linear increases for up to 7 bacteria per host at t0 (**Figure 3E**). For example, the probability of replication of at least one bacterium if two were present at t0 was p_2_ = 0.29 (±0.05), which increased to p_5_ = 0.62 (±0.02) when five bacteria were initially present. We found that the statistical independence model accurately recapitulates the data, with the expected p_0_ = 0.17 (±0.01). This demonstrates that regardless of the number of bacteria per host cell, each bacterium has the same probability to establish a replicative invasion, thus acts independently and non-cooperatively.

We noted that the distribution of number of bacteria associated with host cells at t0 exhibits substantial heterogeneity; 33% of host cells were not infected, while some macrophages harboured up to 15 bacteria (**Figure S4A**). If the bacterial association was due to a purely random process, the distributions of associated bacteria should follow a one parameter Poisson distribution (41). However, the Poisson fit could not capture the data suggesting that a more complex process is involved. Indeed, we found that a negative binomial distribution accurately captures both the increased number of host cells with a very high pathogen number as well as reduced number of those with few or zero bacteria at t0. This suggests that bacterial association with host cells is not purely random, but rather some host cells appear to be more susceptible. We found that the number of adherent bacteria was negatively correlated with the number of neighbouring cells (correlation coefficient R^2^ = 0.47, p-value <0.01, **Figure S4B**), suggesting that adherence is mainly driven by physical accessibility. In the invasion of non-immune cells, specific ligand/receptor interactions between *L. monocytogenes* and the host are required (20), which may explain heterogeneity of cell adhesions observed in endothelium (29). However, in the case of macrophages, *L. monocytogenes* is actively taken up through the host mediated phagocytosis (42). This suggested that at least in our infection experiments, isolated host cells are more likely to be infected by *L. monocytogenes* than those surrounded by neighbours, probably through increased cell surface available for bacteria to bind. While this may be a consequence of our monolayer cell assay, the physical accessibility *in vivo* to *L. monocytogenes* may be governed by complex spatial interactions between macrophages and other cell types (8).

Overall, these analyses indicate that fate of individual *L. monocytogenes* are independent and non-cooperative in the same host, and the ability to replicate is controlled by behaviour of bacteria.

### MOI regulates internalisation but not intracellular replication of *L. monocytogenes*

**Figure 4:**
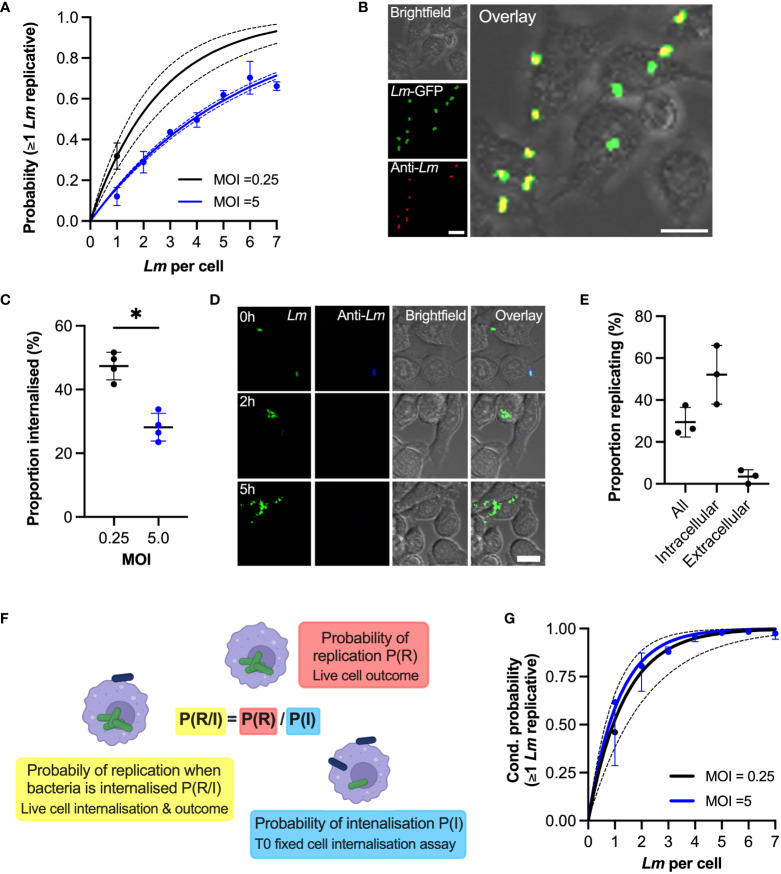
Phagocytosis affects internalisation of bacteria **(A)** Probability of replication depends on MOI. Shown is the probability at least one *L. monocytogenes* replicates as a function of number of bacteria per host cell at t0. Solid black line is the predicted probability (with SDs in broken lines) for MOI 0.25, calculated for multiple invasions per host cell given the replication probability p_0 =_ 0.32 (±0.06) for one bacterium per cell (black circle, from **Figure 1C**). Similarly, in blue the observed (circles denoting mean with SDs) and expected (solid line with broken line SDs) probabilities for MOI 5 (from **Figure 2E**). **(B)** Representative images from internalisation assay showing RAW 264.7 macrophages (brightfield) infected with *Lm*-GFP (green) at MOI 5 fixed at t0 and stained with anti-Lm 594 (red). Scale bar 10 μM. **(C)** Rate of phagocytosis depends on the MOI. The proportion of intracellular *Lm*-GFP at t0 for MOI 0.25 and 5.0 (as a function of the total interactions) from assay depicted in **(B)** Individual data points (circles) from four biological replicates with solid lines indicating mean and SD. Statistical significance (* = p-value <0.05) assessed using Mann-Whitney two sample test. **(D)** Representative images of live-cell infection with *Lm*-GFP internalisation staining and infection outcome tracking. Shown are *Lm*-GFP (green), anti-Lm (blue) and RAW 264.7 (brightfield) infection at MOI 0.25, at indicated times. Scale bar 10 μM. **(E)** Proportion of replicating bacteria based on the internalisation status as depicted in **(D)** Shown is proportion replicating of the total *Lm*-GFP associated with a host cell at t0 (all), proportion of the internalised at t0 (intracellular) or proportion of the bacteria that is associated but are not internalised at t0 (extracellular), evaluated at 5 **(H)** RAW 264.7 macrophages infected with *Lm*-GFP at MOI 0.25 using anti-*Lm* 421 antibody to mark extracellular bacteria at t0. Shown are individual data points (circles) from three replicates with solid lines indicating mean and SD. **(F)** Schematic representation of the conditional probability of replication based on the probability of replication being adjusted to account for the contribution of probability of internalisation. **(G)** Phagocytosis rate explains MOI-specific replication probabilities. Shown are conditional probabilities (of replication given internalisation as described in **(F)** of at least one bacterium replicating as a function of number of bacteria per host cell at t0. Black line indicates the expected probability (mean with SDs) calculated for multiple invasions per host cell given the replication probability p_0 =_ 0.52 ±0.14 (black circle) of internalised bacteria for MOI 0.25 (from **Figure 3E**). Solid blue line depicts expected conditional probabilities assuming statistical independence and a single overall internalisation rate (0.28 ±0.04 from **(C)** based on the probabilities in **Figure 2E**. Blue circles denote conditional probabilities (mean and SDs, based on three replicate experiments) for MOI 5, calculated from data in **Figure 2A** based on the proportion of internalised bacteria in **Figure S3**. .

Our data demonstrate that approximately a third of individual bacteria associated with host cells (referred to here as the overall replication) were able to establish a replicative infection at MOI 0.25 (**Figure 1C**). Surprisingly, when the infection was performed at MOI 5 the overall replication probability was reduced by 2-fold (**Figure 4A**). Specifically, the probability of replication when one bacterium interacted with a host cell (p_1_) was reduced from 0.32 (±0.06) for MOI 0.25 to 0.17 (±0.01) for MOI 5, while the expected probabilities as a function of number of interacting bacteria exhibited statistically non-overlapping trends. These data demonstrate that changing the MOI affects the overall replication probability.

In order to replicate in macrophages bacteria must enter the host cell through phagocytosis and escape to the cytoplasm (21). To test how MOI affects replication probability, we used anti-*L. monocytogenes* (anti*-Lm*) antibody staining to distinguish bacteria that were internalised from those that were adhering to the cell surface in cells fixed at t0 (**Figure 4B**). The proportion of internalized bacteria at t0 at MOI 5 was 28.12 ±4.4% (based on 749 cells that had associated bacteria at t0), which is in good agreement with the previously published 25% internalisation rate in RAW 264.7 cells at MOI=10 (43). In comparison, 47.4 ± 4.3% of bacteria were internalised at MOI 0.25 (based on 952 cells) demonstrating that higher MOI reduced the rate of phagocytosis (**Figure 4C**).

Subsequently, we developed a live cell version of the internalisation assay, where we followed *Lm-*GFP after live-cell anti-*Lm* extracellular staining at t0, allowing us to determine the fates of internalised vs. extracellular bacteria (**Figure 4D**; **Video 5**). We found that for MOI 0.25 (for which individual bacteria can be tracked over time) 52% (±14%) of bacteria that were internalized at t0 replicated, while only 3.5% (±1.9%) of the bacteria that were extracellular at t0 replicated by 5h (**Figure 4E**). Due to gentamicin-mediated killing of extracellular bacteria (44), we predict the latter likely correspond to bacteria that are in the process of internalisation at t0. We then introduced conditional probabilities to simplistically characterise the two-step internalisation/replication process, such that the conditional probability of replication given that a bacterium is internalised (probability of replication of intracellular bacteria) P(R/I)=P(R)/P(I) is the ratio of the overall replication probability P(R) and the probability of internalisation P(I) (**Figure 4F**). According to this model for MOI 0.25, the expected conditional probability of replication given that one bacterium is internalised was P(R/I)=0.62, based on the measured overall replication probability P(R)=0.29 (**Figure 4E**) and the probability of internalisation P(I)=0.47 (**Figure 4C**). Therefore, the expected probabilities obtained using fixed cell internalisation assay and observed conditional proportions based on dual live-cell staining assay are in the good agreement.

Having confirmed the conditional probability model, we next examined whether the change of the internalisation rate might explain apparent differences in probability of replication observed at different MOIs. The conditional probabilities that at least one bacterium replicates calculated for MOI 0.25 and MOI 5 based on the associated internalisation rates (**Figure 4C**, see Materials and Methods for derivations) followed very close trends (**Figure 4G**, black solid line vs. blue line). While initially we assumed the overall internalisation rate for MOI 5, we additionally examined the proportion of internalised bacteria as a function of number of bacteria at t0 (**Figure S5**). We found a small but significant linear increase from 26.4 (±1.3%) when one bacterium is present up to 41.6 (±5.7%) when seven bacteria were present (R^2^ = 0.27, p-value 0.01), suggesting that increased number of bacteria may increase rate of phagocytosis or, according to our previous finding, isolated cells exhibit higher rate of phagocytosis (**Figure S4B**). Nevertheless, the conditional probabilities that at least one bacterium replicates calculated for MOI 0.5 based on the internalisation rates calculated as function of bacteria per cell (**Figure 4G**, blue circles) were also in a good agreement with conditional probabilities for MOI 0.25. Therefore, these analyses demonstrate that the changes of the overall replication probability in response to MOI is controlled through the rate of the phagocytosis, while the replication probability of intracellular bacteria was unaffected (52% vs 59% for MOI 0.25 vs 5, respectively, according to the fitted model).

### Secreted LLO levels regulate *L. monocytogenes* internalisation

**Figure 5:**
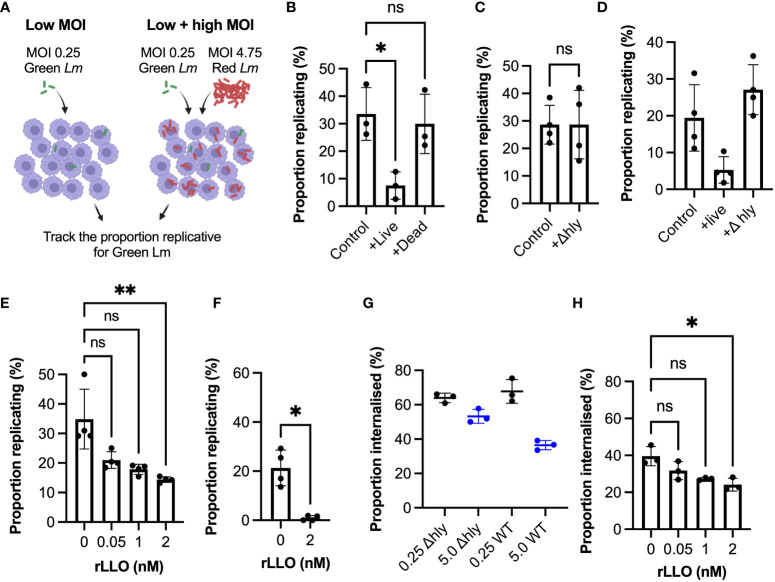
Listeriolysin O controls infection outcomes at the population-level **(A)** Schematic representation of experimental set up for infections with *Lm*-GFP and *Lm*-mCherry strains at combined MOI 5 vs *Lm*-GFP MOI 0.25 control. **(B)** Proportion of *Lm*-GFP MOI 0.25 replicating upon infection of RAW 264.7 macrophages for *Lm-*GFP only (control), or for *Lm*-GFP when live (+live) or PFA fixed (+dead) *Lm*-mCherry added for combined MOI 5 (as a function of the total *Lm*-GFP interactions). Triplicate data (circles) with mean and SD (solid lines). Statistical significance assessed using Kruskal-Wallis ANOVA with Dunn’s correction for multiple comparisons (ns = non-significant, * = p-value<0.05). **(C)** Proportion of *Lm*-GFP (MOI 0.25) replicating upon infection of RAW 264.7 macrophages for *Lm-*GFP only (control), or for *Lm*-GFP when Δ *Lm*-mCherry (+Δ*hly*) added for combined MOI 5 (as a function of the total *Lm*-GFP interactions). Replicate data from four experiments (circles) with mean and SD (solid lines). Statistical significance assessed with Mann-Whitney rank test (ns = non-significant). **(D)** Proportion of *Lm*-GFP replicating upon infection of BMDMs at MOI (0.25) (control), or when live WT *Lm*-mCherry (+live) or Δ*hly Lm*-mCherry (+Δ*hly*) added for combined MOI 5 (as a function of the total *Lm*-GFP interactions). Data from four experiments (circles) with mean and SD (solid lines). Statistical significance assessed using Kruskal-Wallis ANOVA with Dunn’s correction for multiple comparisons (ns = non-significant, * = p-value<0.05). **(E)** Proportion of *Lm*-GFP replicating upon infection of RAW 264.7 macrophages at MOI 0.25 (as a function of the total interactions at t0), with addition recombinant listeriolysin (rLLO). Individual data from four experiments (circles) with mean and SD (solid lines). rLLO at indicated concentrations added with inoculant and removed at t0. Statistical significance assessed using Kruskal-Wallis ANOVA with Dunn’s correction for multiple comparisons (ns = non-significant, ** = p-value<0.01). **(F)** Proportion of *Lm*-GFP replicating upon infection of BMDM at MOI 0.25 (as a function of the total *Lm*-GFP interactions), incubated with 0 or 2 nM rLLO. Individual data from three experiments (circles) with mean and SD (solid lines). rLLO added with inoculant and removed at t0. Statistical significance assessed with Mann-Whitney rank test (* = p-value<0.05). **(G)** Proportion of WT *Lm*-GFP and Δ*hlyLm*-GFP internalised into RAW 264.7 macrophages at MOI 0.25 and 5 (as a function of the total interactions). Data obtained from internalisation assay using anti-*Lm* 594 staining of infected cells fixed at t0 as depicted in 3b. Values from triplicate data (circles) with mean and SD (solid lines). Statistical significance assessed using Kruskal-Wallis ANOVA with Dunn’s correction for multiple comparisons (ns = non-significant, * = p-value<0.05). **(H)** Proportion of *Lm*-GFP internalised into RAW 264.7 macrophages at MOI 0.25 (as a function of the total *Lm*-GFP interactions) in the presence of recombinant LLO (rLLO). Individual replicate data from four experiments (circles) with mean and SD (solid lines), from internalisation assays using anti-*Lm* 594 staining of infected cells fixed at t0. rLLO concentration indicated on the graph. Statistical significance assessed using Kruskal-Wallis ANOVA with Dunn’s correction for multiple comparisons (ns = non-significant, * = p-value<0.05).

To mechanistically understand how the overall replication probability depends on the different number of bacteria in the environment we devised a dual colour experiment where *Lm*-GFP (green *Lm*) equivalent of MOI 0.25 was supplemented with *Lm*-dsRed such that the overall MOI was maintained at 5 (**Figure 5A**). We found that supplementation with live *Lm*-dsRed significantly reduced the proportion replicating bacteria, while supplementation with PFA fixed (dead) *Lm*-dsRed had no significant effect in comparison to the control *Lm*-GFP at MOI 0.25 (**Figure 5B**). This suggests a role for a secreted factor produced by bacteria, which is consistent with the soluble pore-forming toxin LLO (45). Indeed, we found when WT *Lm*-GFP were supplemented with *Δhly Lm*-dsRed, unable to produce LLO, the replication probability was not affected at an MOI of 5 (**Figure 5C**). Replication probability was similarly affected by live WT but not *Δhly L. monocytogenes* upon invasion of BMDMs (**Figure 5D**).

LLO is known to play multiple roles during invasion, including activation of host immune responses (45–48), we therefore tested whether recombinant LLO alone can inhibit *L. monocytogenes* replication and internalisation. Indeed, we found that incubation with previously used non-cytolytic doses of recombinant LLO (49) significantly reduced replication at MOI=0.25 (**Figure 5E**), without affecting cell membrane permeability and viability (**Figure S6**). In BMDMs LLO treatment almost completely prevented replication, with <1% bacteria able to establish replicative invasions (**Figure 5F**). Consistent with a role for phagocytosis, we observed limited changes of internalisation of *Δhly* strain at MOI 0.25 vs 5, in contrast to the WT bacteria (**Figure 5G**). Finally, we showed that treatment with recombinant LLO also significantly reduced the internalisation of bacteria (**Figure 5H**). Overall, these data demonstrate that the amount of LLO in the environment surrounding the host macrophages regulates the overall *L. monocytogenes* replication by controlling bacterial uptake.

## Discussion

Interactions between host and pathogen at the single cell level are inherently heterogeneous leading to different infection outcomes. Here we use time-lapse confocal microscopy to follow with a single bacterium resolution the fate of the food-borne pathogen *L. monocytogenes* upon invasion of innate immune macrophages, a key event controlling the overall infection. We demonstrate that invasion of macrophages results in heterogeneous outcomes, where only a fraction of single-cell host pathogen interactions leads to intracellular replication and spread of bacteria, while many bacteria are cleared or remain in a non-replicative state in the host (**Figure 1**).

In our datasets focusing on murine macrophages ~30% of bacteria associated with the host were able to establish replicative infection, both in RAW 264.7 line as well as primary BMDMs. Successful replication of *L. monocytogenes* is a muti-step process requiring host cell attachment, internalization into a phagosome, phagosome escape and cytosolic replication (20). When we simplistically described this process using conditional probabilities accounting for intracellular replication based on internalisation and the overall replication, we found that, after internalisation by a host cell, approximately 50% of intracellular bacteria can establish a replicative infection, regardless of the level of infection (i.e., low or high MOI, **Figures 3**, **4**). At low MOI, we found that approximately 50% of bacteria are internalised, while approximately at least 80% of intracellular bacteria escape phagosome based on positive actin and LAMP1 staining (**Figure 1**). Furthermore, 75% of cytoplasmic bacteria can replicate, while 25% of those (equivalent of 11% of total bacteria) persist in the cytoplasm in a non-replicative state for up to 5 h. This is in good agreement with previously published 25% internalisation rate in RAW 264.7 cells at MOI=10 (43) as well as 55% in BMDMs (50) and 60-70% in primary dendritic cells (51) across a range of MOIs. However, the rate of phagocytosis generally varies across phagocyte subsets, with murine monocytes being particularly resistant to *L. monocytogenes* invasion *in vivo* (<1% internalisation rate) (51). In contrast, the rate of phagosomal escape appears to be conserved around 75-90% across different macrophages and experimental settings (32, 35, 52) (33, 51).

Previous measurements of replication have used gentamicin protection assays that do not follow individual bacteria over time, but merely enumerate the number of viable/intracellular bacteria at different time points, and as such are likely unable to identify non-replicative bacteria. However, one study, which similarly followed fate of individual bacteria in the non-phagocytic human cells for up to 72 h showed that during prolonged invasion the intracellular *L. monocytogenes* may switch between replicative and a persistent non-replicative state (28). In our analyses, we focused on the initial phase of the infection, when within the 5h window non-replicative bacteria and replicative foci could be accurately distinguished, while providing sufficient sample size per replicate. Our imaging experiments provide no evidence for the re-activation of the persistent cells (within the 5 h time window), instead the replication occurs as quickly as 30 mins after addition of inoculum, with almost all cells establishing replication within 2 h window post infection. These may represent differences in the intracellular environment between macrophages used in our study and non-immune cells used previously including timing of lysosome and phagosome fusion, depending of their availability in different cells, or ability of individual *L. monocytogenes* to resist lysosomal acidification (28). On the other hand, the delayed replication or in fact re-activation of the persistent bacteria may possibly occur in macrophages beyond the 5 h time window, which would be relevant to investigate in the future. While here we focused on murine macrophages, further investigation of the described phenomenon upon human macrophage infection would be important.

Which pathway in *L. monocytogenes* controls the ability to establish successful replication remains unclear, but certainly phagosome escape is not the only control mechanism, given that our data demonstrate ~11% of all bacteria are cytoplasmic, but do not replicate (**Figure 1**). While our data demonstrate that PrfA activity itself does not control replication, other factors may contribute to this process, for example the DNA uptake competence (Com) system, controls phagosome escape and exhibits expression variability consistent with heterogenous outcomes (35, 53). It is known that the physiological status of the bacterium is important for virulence, with crosstalk between the metabolism and virulence in part mediated *via* CodY (54). In turn proliferation has been also shown to depend on the nutrients acquired from the host, for example uptake of microsomal glucose *via* hexo phosphatase transporter (Hpt) (55), which although PrfA-dependent in principle, might exhibit heterogenous expression or depend on the variable intracellular supply in the host cell.

Pathogens often cooperate to overcome host cell defences (56). For example, cooperativity between *Salmonella* allows non-invasive strains to enter host cells (57–60). Once in the host, cooperativity among bacterial effector protein enable suppression of the immune defences by targeting multiple signalling responses (61). In turn, host cells use collective behaviour, including quorum-like activation of their signalling responses, to enhance immune responses (62) for better pathogen control (63). Using dual colour experiments we found that the ability of individual *L. monocytogenes* to replicate in the same host cell are independent from the host cell and non-cooperative (**Figure 3**). Specifically, when multiple bacteria invade the same host cell, each bacterium acts independently with the same probability of replication. Importantly, while not providing any apparent advantage, the presence of multiple bacteria in the same host cell virtually assures replication and subsequent rapid intracellular proliferation of *L. monocytogenes*. For example, the probability of replication for >3 bacteria per host exceeds 90%. Paradoxically, we found that higher MOI resulted in ~2-fold reduction of the overall replication probability. We were able to demonstrate that this was due to expression of the PrfA-regulated LLO, a pore forming toxin, at high MOI. LLO alone was sufficient to inhibit phagocytosis and overall replication in LLO treated cells (**Figure 5**), without affecting cell membrane permeability (**Figure S6**). At the single cell level, we show intracellular upregulation of PrfA activity, which is necessary for phagosome escape (38), does not alter the intracellular replication of intracellular bacteria (**Figure 2**). Our data reveals a population-level strategy where bacterial populations control the overall invasion outcome *via* coordinated LLO secretion, but once inside a host cell individual bacteria act independently and non-cooperatively, with respect to PrfA activity and in general.

Our data suggest that LLO secretion and reduced phagocytosis *in vivo* might assure successful bacterial replication while simultaneously increasing the likelihood of systemic dissemination through the blood stream, and uptake by non-phagocytic cells (64) to promote immune evasion. LLO is a pore-forming toxin, pH-dependent member of the cholesterol-dependent cytolysins (CDCs) which binds cholesterol present in the host cell membrane (45). It is necessary for the vacuolar escape of *L. monocytogenes*, but plays many other roles, including control of autophagy and mitophagy (48), and suppression of ROS production (47), but also activates host signalling responses (46). Previous work suggests that the formation of LLO pores at the cell membrane increases *L. monocytogenes* internalisation into non-phagocytic cells (64, 65). Our data demonstrate that in macrophages, the rate of phagocytosis decreased upon LLO exposure, while the effect on overall ability to establish replicative invasion, especially in primary macrophages is substantial. Phagocytosis was previously shown to be regulated, in part through p38 mitogen activated protein kinase, in response to TRL2-dependent Gram-positive *Staphylococcus aureus* (66, 67) and *L. monocytogenes* (50). Phagocytosis of *L. monocytogenes* has also been linked to the expression of the inhibitory receptor T-cell immunoglobin mucin-3 (Tim-3), an immune checkpoint inhibitor (68). Tim-3 inhibits the rate of phagocytosis by inhibiting expression of the CD36 scavenger receptor (43), which is involved in phagocytosis of Gram positive bacteria (69). Tim-3 itself and its ligand Galectin-9 both have been shown to be upregulated by infection (70). In addition, pneumolysin and other CDCs including LLO have been shown to bind the mannose receptor (MCR1), while blocking MRC1 resulted in reduced uptake and intracellular survival of *Streptococcus pneumoniae* (49). It is currently unclear, whether physiological levels of LLO may indeed be sufficient to alter the expression of these receptor system and thus alter phagocytosis. Nevertheless, these data suggest that control of phagocytosis *via* CDCs might represent an important invasion strategy for bacterial pathogens. In agreement, pneumonolysin, which is structurally similar to LLO, was also shown to inhibit phagocytosis of *S. pneumoniae* in neutrophils (71). In turn, the control of phagocytosis and phagosome maturation remains an important host defense strategy (72–76), highlighting the critical role of phagocytosis in host pathogen interactions.

Overall, our analyses reveal new insight into distinct single cell and population-level strategies of *L. monocytogenes* upon invasion of innate immune macrophages. We demonstrate that while inside the host cells, individual bacteria act independently and non-cooperatively, the overall bacterial population control outcomes of single cell host interactions through PrfA signalling and LLO secretion.

## Materials and methods

### Bacterial strains culture conditions

*L. monocytogenes* EGDe : InlA^m^ (77) was used as the wild type (WT), with all mutations generated in this background. *L. monocytogenes* was grown in tryptone soya broth (TSB) unless otherwise stated, when needed antibiotics were added at final concentrations of: chloramphenicol (Cm) 7 μg ml^-1^ and erythromycin (Em) 5 μg ml^-1^. *Escherichia coli* DH5α was used for cloning and grown in Luria-Bertani broth (LB), when needed antibiotics were added at final concentrations of: chloramphenicol (Cm) 35 μg ml^-1^ and erythromycin (Em) 150 μg ml^-1^. Viability of stored bacteria was confirmed by routine measurements of colony forming units of the inoculum.

Plasmids (**Table 1**) were electroporated into *L. monocytogenes* to generate fluorescently tagged and fluorescent reporter strains of *L. monocytogenes* described in the same table. Chromosomal integration of integrative plasmids was confirmed by PCR as described previously (81). Correct fluorescence of strains was confirmed by microscopy.

**Table 1:** Plasmids and strains used in this study.

Plasmid, strain or primer name	Description	Antibiotic resistance	Reference
Plasmids			
pAD_1_-cGFP	Integrative plasmid with constitutive GFP expression	Cm	(78)
pAD_3_-P*actA*-GFP	Integrative plasmid expressing GFP under control of P*actA*	Cm	(78)
pCG8	Integrative plasmid expressing codon optimised GFP under control of P*hly*	Cm	(26)
pJEBAN6	Plasmid expressing constitutive DsRedExpress	Em	(79)
pPL2-mCherry	Integrative plasmid expressing constitutive codon optimised mCherry	Cm	(80)
Bacterial strains			
EGDe : InlA^m^	EGDe strain with murinized InlA protein	–	(77)
Δ*hly Lm*	EGDe : InlA^m^ *hly* deletion mutant	–	(22)
Δ*prfA Lm*	EGDe : InlA^m^ *prfA* deletion mutant	–	This study
PrfA* *Lm*	EGDe : InlA^m^ PrfA* mutant	–	This study
*Lm*-GFP	EGDe : InlA^m^ with integrated pAD_1_-cGFP	Cm	This study
*Lm*-DsRed	EGDe : InlA^m^ with pJEBAN6	Em	This study
*Lm*-mCherry	EGDe : InlA^m^ with integrated pPL2-mCherry	Cm	This study
Δ*hly Lm*-GFP	Δ*hly Lm* with integrated pAD_1_-cGFP	Cm	This study
Δ*hly Lm*-mCherry	Δ*hly Lm* with integrated pPL2-mCherry	Cm	This study
*Lm*-dsRed P*actA*-GFP	EGDe : InlA^m^ with integrated pAD_3_-P*actA*-GFP and pJEBAN6	Cm, Em	This study
*Lm*-dsRed P*hly*-GFP	EGDe : InlA^m^ with integrated pCG8 and pJEBAN6	Cm, Em	This study
PrfA* *Lm*-GFP	PrfA* *Lm* with integrated pAD_1_-cGFP	Cm	This study
Δ*prfA Lm*-dsRed P*hly*-GFP	Δ*prfA Lm* with integrated pAD_3_-P*actA*-GFP and pJEBAN6	Cm, Em	This study
PrfA* *Lm*-dsRed P*hly*-GFP	PrfA* *Lm* with integrated pAD_3_-P*actA*-GFP and pJEBAN6	Cm, Em	This study

*L. monocytogenes* PrfA* and Δ*prfA* mutants were constructed using the temperature sensitive shuttle plasmid pAUL-A as described previously (22).

### Cell culture

RAW 264.7 macrophages were maintained in Dulbecco’s modified Eagle medium (DMEM) supplemented 10% (v/v) fetal bovine serum (FBS) and 1% (v/v) 100x MEM non-essential amino acid solution (NEAA) at 37 °C 5% (v/v) CO_2_. Bone marrow derived macrophages (BMDMs) were generated from C57BL/6 female mice using L929-conditioned media, once differentiated BMDMs were maintained for up to 3 days in DMEM supplemented with 10% (v/v) FBS at 37 °C 5% (v/v) CO_2_. All animal procedures were performed with appropriate personal and project licenses in place, in accordance with the Home Office (Animals) Scientific Procedures Act (1986), and approved by the Home Office and the local Animal Ethical Review Group, University of Manchester. Cell membrane permeability and viable cell count was measured with haemocytometer (Merck) using 0.4% (w/v) Trypan Blue (ThermoFisher) staining as described (82).

### Live-cell microscopy infection assays

RAW 264.7 macrophages or BMDMs were seeded in 35 mm TC treated imaging dishes (Cellview Greiner) at 3.5x10^5^ and 7x10^6^ cells ml^-1^ respectively and incubated overnight. *L. monocytogenes* mid-log (OD_600_ 0.45-0.6) aliquots stored at -80 °C in PBS glycerol (15% v/v) were used for infections. Cells were infected with *L. monocytogenes* at a MOI of 0.25 in pre-warmed media for 45 min and washed three times prior to the addition of 10 μg ml^-1^ gentamicin media (**Figure 1A**). For assays with recombinant listeriolysin (rLLO, Abcam), rLLO was added to cells with the *L.monocytogenes* at a final concentration of 0.05-2 nM. Infections were immediately imaged by live-cell time-lapse microscopy using a Zeiss LSM710, Zeiss LSM780 or Zeiss LSM880 microscope. Data was visualised using the Zeiss Zen Black software.

### Bacterial staining and internalisation assays

For internalisation and actin staining assay infections were performed as described above but fixed at 0 h and 5 h respectively with 4% (w/v) PFA PBS for 30 min at room temperature. For the internalisation assay, extracellular *L. monocytogenes* were stained with polyclonal rabbit anti-listeria (anti-*Lm*) antibody (Abcam, ab35132), and washed 3 times before secondary antibody staining with anti-rabbit IgG 594 (Sigma-Aldrich). For the live-cell internalisation and infection outcome assay, anti-*Lm* was added to live cells in pre-warmed media for 30 s, washed 3 times before secondary antibody staining with Brilliant violet 421 donkey anti-rabbit antibody (Biolegend) in pre-warmed media for 30 s, and washed 3 times before imaging.

For the actin staining assay fixed cells were permeabilised 0.1% triton X-100 (v/v) PBS for 4 min and washed 3 times with PBS. As permeabilization sometimes affected *Lm*-GFP signal intensity, anti-*Lm* staining was then performed as described above, but with anti-rabbit IgG 488 (Biolegend) secondary antibody. Alexa fluor 594 Phalliodin (ThermoFisher) was used to stain actin.

For lysosomal staining, cells were fixed using 4% (w/v) PFA for 15 minutes before washing with PBS. Cells were then permeabilised with 0.1% Triton X-100 (v/v) in PBS with 1% (w/v) BSA and incubated for an hour in the presence of anti-LAMP1 (Biolegend, cat no. 121622) conjugated with Alexa Fluor 594, followed by a final wash with PBS and 1% (w/v) BSA.

### Analysis of imaging data

To identify infection outcomes in time-lapse microscopy data, individual *L. monocytogenes* visible at t0 were visually tracked in Zen Black software and recorded as bacterial replication, no-replication or bacteria disappear (**Figure 1B**). Correlations between actin or anti-*Lm* staining were manually assessed in Zen Black. Co-localisation of anti-LAM and LAMP1 staining was assessed in Cell Profiler (83). Evidence of single/multiple bacteria per host cells was used to stratify non-replicating/replicating bacteria, respectively.

For tracked *L. monocytogenes* GFP reporter expression (P*hly* or P*actA*) during infection, individual *L. monocytogenes* were highlighted as regions of interest for selected time points in FIJI (84), and relative fluorescence intensities (RFU) exported for downstream analysis.

For *L. monocytogenes* P*hly*-GFP reporter expression in media at selected timepoints, automated analysis of exported tif images was performed in CellProfiler (83). Brightfield images were used to segment images and identify bacterial cell outlines, relative fluorescence intensities for individual bacteria were then exported for downstream analysis.

### Analysis of replication probabilities

In general, the conditional probability that bacteria replicates (R) given that it is internalised (I); P(R/I), can be expressed as the ratio of the overall replication probability P(R) and the internalisation probability P(I); such that P(I)=P(R)/P(I). Then P_n_(I) = P_n_(R)/P_n_(I) denotes respective probabilities for n bacteria adhering to the host cell at t0. The expected conditional probability that at least one bacteria replicates is given by p_n_(R/I)=1-(1- P_n_(R/I))^n^, where in general P_n_(R/I) may depend on n. However, under the statistical independence model (**Figure 2D**), these relationships are equivalent to p_n_(R/I)=1-(1- p_0_(R/I))^n^ where n is the number of adherent bacteria and p_0_(R/I)=I(R/I)= P_1_(R)/P_1_(I) is the probability of replication if one bacteria is present. For MOI 0.25, p_0_(R/I) was measured directly using live-cell microscopy with additional staining (**Figure 3D**), and subsequently used to calculate expected probabilities for n>1 (**Figure 3G**, blue curve). For MOI 5, we used a previously fitted p_0 =_ 0.164 (**Figure 2E**) such that p_0_=P_n_(R) and p_n_(R/I)=1-(1- p_0_/P_n_(I))^n^. Then the probability of internalisation P_n_(I) was either measured for each n (**Figure S3**) or a single average rate was used (as in **Figure 3C**).

### Statistical analyses

Statistical analysis was performed using GraphPad Prism 8 software (version 8.4.2). The D’Agostino-Pearson test was applied to test for normal (Gaussian) distribution of acquired data. Two-sample comparison was conducted using non-parametric Mann Whitney test, for analyses of variance Kruskal-Wallis ANOVA with Dunn’s multiple comparisons test was performed. Simple linear regression and Pearson’s correlation coefficient R^2^ was used to test association between two selected variables. Schematic Displays were created with BioRender.com.

## Data availability statement

The raw data supporting the conclusions of this article will be made available by the authors, without undue reservation.

## Ethics statement

The animal study was approved by the Home Office and the local Animal Ethical Review Group, University of Manchester. The study was conducted in accordance with the local legislation and institutional requirements.

## Author contributions

JM developed bacterial strains for imaging, collected, and analysed data. JB provided preliminary data for the project and performed fixed cell staining. MG and EL assisted with strain development. LF, CN and DS assisted with imaging analyses. IR and PP provided supervision and conceptualisation, and with assistance of JM wrote the manuscript. All authors contributed to the article and approved the submitted version.

## Supplementary material

Supplementary Figure 1 Analysis of LAMP1 staining upon *Lm* infection **(A)** Representative image of actin staining showing: *Lm-*GFP replicating and no associated with LAMP1(arrow pointing up) and non-replicating associated with LAMP1 (arrow pointing down). RAW 264.7 macrophages infected with *Lm*-GFP were fixed at 5 h, permeabilised then stained with anti-*Lm* (green) and LAMP1-594 (magenta). Scale bar 10 μM. Images representative of three replicated experiments. **(B)** Quantification of *Lm* positive (in green) and negative (in grey) host cells across data from a. **(C)** Quantification of replicative (in yellow) and non-replicative (in grey) across *Lm* positive host cells from b).

Supplementary Figure 2 PrfA operon reporter expression at the single cell level. **(A)** Representative images of live-cell WT, Δ*prfA* or PrfA** Lm*-dsRed P*hly*-GFP incubated in DMEM over time. Shown are *Lm* cells (brightfield) and expression of GFP under the control of the *hly* promoter region (GFP) at 0.5, 1.5 or 2.5h after addition of *Lm*. Scale bar 5 μM. **(B)** GFP fluorescence expression from the *hly* promoter over time. Data from 3 replicate experiments (minimum 137 total individual cells per condition) for WT (pink), Δ*prfA* (black) or PrfA*** (teal) *Lm*-dsRed P*hly*-GFP incubated in DMEM. Automated cell identification and GFP intensity measured as relative fluorescence units (RFU) in Cell Profiler at 0.5, 1.5 or 2.5h after addition of *L. monocytogenes* to media. Statistical significance (ns = non-significant, **** = p-value <0.0001) assessed using Kruskal-Wallis ANOVA with Dunn’s correction for multiple comparisons.

Supplementary Figure 3 Distribution of *L. monocytogenes* replication times post invasion. Shown is the time to first replication of *Lm*-dsRed P*actA*-GFP and *Lm*-dsRed P*hly*-GFP during infection of RAW 264.7 macrophages at MOI 0.25 from pooled data in . Data from 3 replicates and 22 total replicative *L. monocytogenes* presented as a bar graph with mean and SD.

Supplementary Figure 4 Distribution of bacteria adhering to host cells at MOI 5. **(A)** Distribution of bacteria per cell at t0 when RAW 264.7 macrophages simultaneously infected with *Lm*-dsRed and *Lm*-GFP at combined MOI 5 (at 1:1 ratio). Plot shows mean frequency of distribution for experimental data (black) from 3 replicates, and expected data assuming a one parameter Poisson distribution (pink) or negative binomial distribution (teal). **(B)** Relationship between mean frequency of bacteria per cell at t0 and number of neighbouring cells when RAW 264.7 macrophages simultaneously infected with *Lm*-dsRed and *Lm*-GFP at combined MOI 5 (at 1:1 ratio). Data from 2 replicates (black circles) and simple linear regression (black line, R^2^ = 0.47, p-value = 0.007).

Supplementary Figure 5 Analysis of *L. monocytogenes* internalisation at MOI 5. Relationship between proportion of internalised *Lm*-GFP and number of *Lm*-GFP associated with the cell at t0 when RAW 264.7 macrophages infected at MOI 5. Individual data from four replicates (circles) and simple linear regression (solid line, R^2^ = 0.27, p-value = 0.01) shown. Data obtained from internalisation assay using anti-Lm 594 staining of infected cells fixed at t0 as depicted in 3b.

Supplementary Figure 6 Analysis of host cell viability upon LLO exposure. Proportion of live RAW 264.7 cell staining incubated with 0 or 2 nM rLLO. Individual data from three experiments (circles) with mean and SD (solid lines). rLLO added with inoculant and removed at t0. Statistical significance assessed with Mann-Whitney rank test (ns -not significant).

Supplementary Video 1 Representative live-cell microscopy of RAW 264.7 macrophages (brightfield) infected with *Lm-*GFP (green) from times 0-10 h post gentamicin treatment. Scale 20μM.

Supplementary Video 2 Representative images of live-cell *Lm*-dsRed P*actA*-GFP (red) infection of RAW 264.7 macrophages (brightfield) at MOI 0.25 from times 0-5 h post gentamicin treatment. GFP expression is driven from the *actA* promoter region (green). Scale bar 10 μM.

Supplementary Video 3 Representative images of live-cell *Lm*-dsRed P*hly*-GFP (red) infection of RAW 264.7 macrophages (brightfield) at MOI 0.25 from times 0-5 h post gentamicin treatment. GFP expression is driven from the *hly* promoter region (green). Scale bar 10 μM.

Supplementary Video 4 Representative live-cell microscopy of RAW 264.7 macrophages (brightfield) simultaneously infected with *Lm*-dsRed (red) and *Lm*-GFP (green) at combined MOI 5 (at 1:1 ratio) from times 0-5 h post gentamicin treatment. Scale bar 10 μM.

Supplementary Video 5 Representative live-cell microscopy of RAW 264.7 macrophages (brightfield) infected with *Lm*-GFP (green) with anti-*Lm* 421 internalisation staining (magenta) from times 0-5 h post gentamicin treatment. Scale bar 10 μM.

Supplementary file 1 Tabularized manuscript data.
