# Supplementary figures and images for: Live-cell imaging reveals single-cell and population-level infection strategies of Listeria monocytogenes in macrophages
Source: Front Immunol. 2023 Aug 22;14:1235675. doi: 10.3389/fimmu.2023.1235675 (PMC10478088; doi:10.3389/fimmu.2023.1235675)

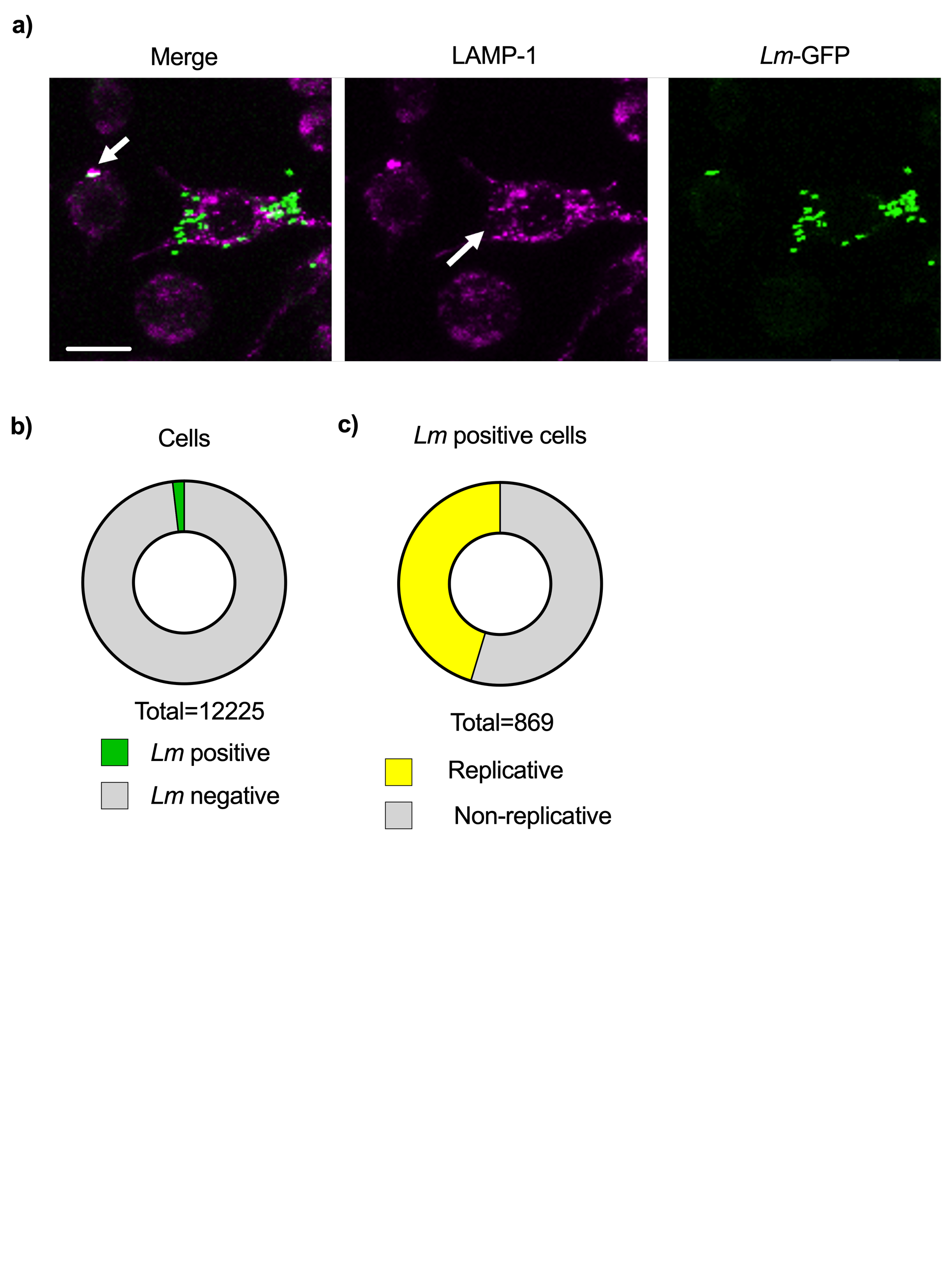

Supplement: Supplementary Figure 1 — Analysis of LAMP1 staining upon Lm infection (A) Representative image of actin staining showing: Lm-GFP replicating and no associated with LAMP1(arrow pointing up) and non-replicating associated with LAMP1 (arrow pointing down). RAW 264.7 macrophages infected with Lm-GFP were fixed at 5 h, permeabilised then stained with anti-Lm (green) and LAMP1-594 (magenta). Scale bar 10 μM. Images representative of three replicated experiments. (B) Quantification of Lm positive (in green) and negative (in grey) host cells across data from a. (C) Quantification of replicative (in yellow) and non-replicative (in grey) across Lm positive host cells from b). [file Image_1.tiff]

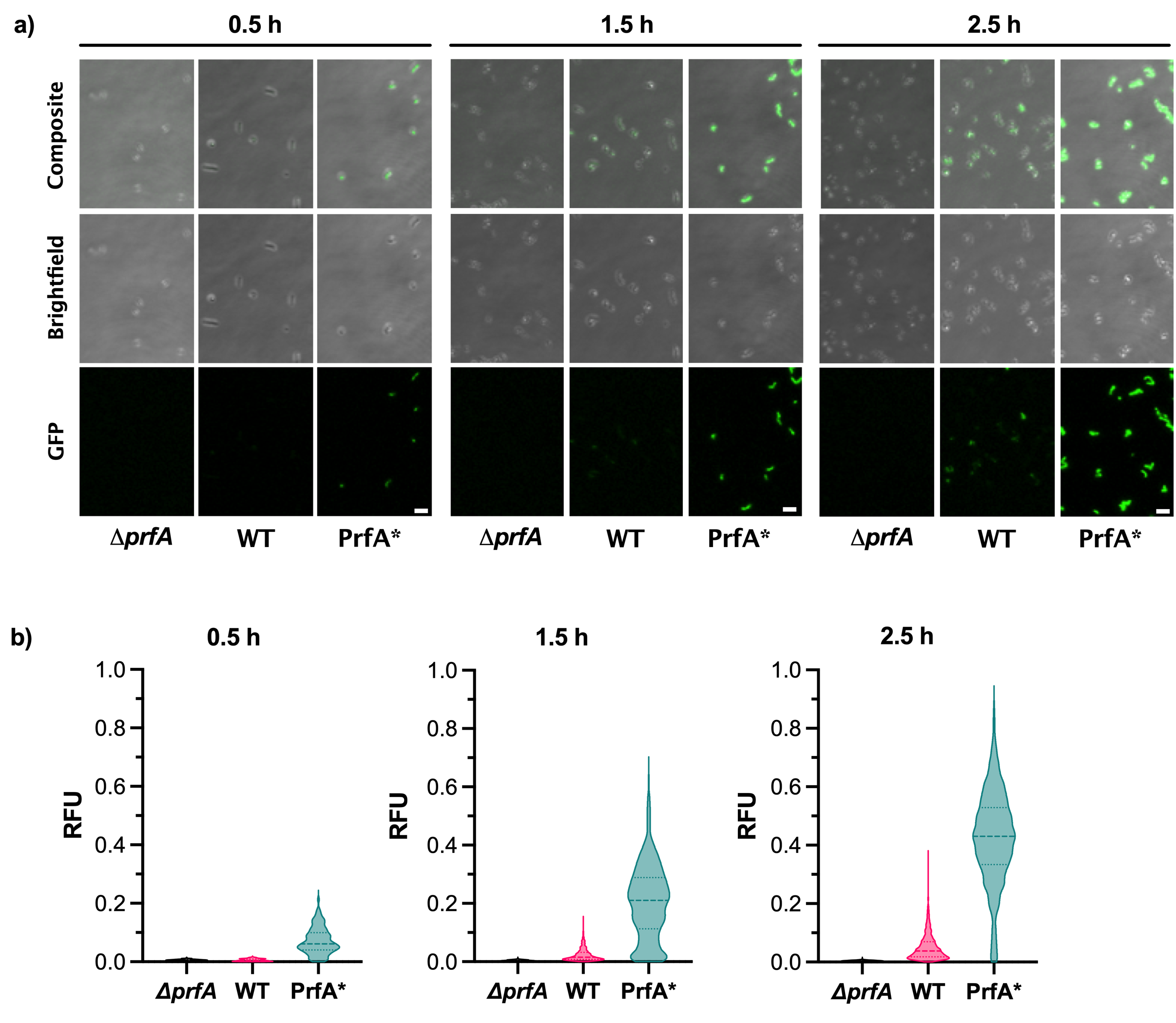

Supplement: Supplementary Figure 2 — PrfA operon reporter expression at the single cell level. (A) Representative images of live-cell WT, ΔprfA or PrfA* Lm-dsRed Phly-GFP incubated in DMEM over time. Shown are Lm cells (brightfield) and expression of GFP under the control of the hly promoter region (GFP) at 0.5, 1.5 or 2.5h after addition of Lm. Scale bar 5 μM. (B) GFP fluorescence expression from the hly promoter over time. Data from 3 replicate experiments (minimum 137 total individual cells per condition) for WT (pink), ΔprfA (black) or PrfA* (teal) Lm-dsRed Phly-GFP incubated in DMEM. Automated cell identification and GFP intensity measured as relative fluorescence units (RFU) in Cell Profiler at 0.5, 1.5 or 2.5h after addition of L. monocytogenes to media. Statistical significance (ns = non-significant, **** = p-value <0.0001) assessed using Kruskal-Wallis ANOVA with Dunn’s correction for multiple comparisons. [file Image_2.tiff]

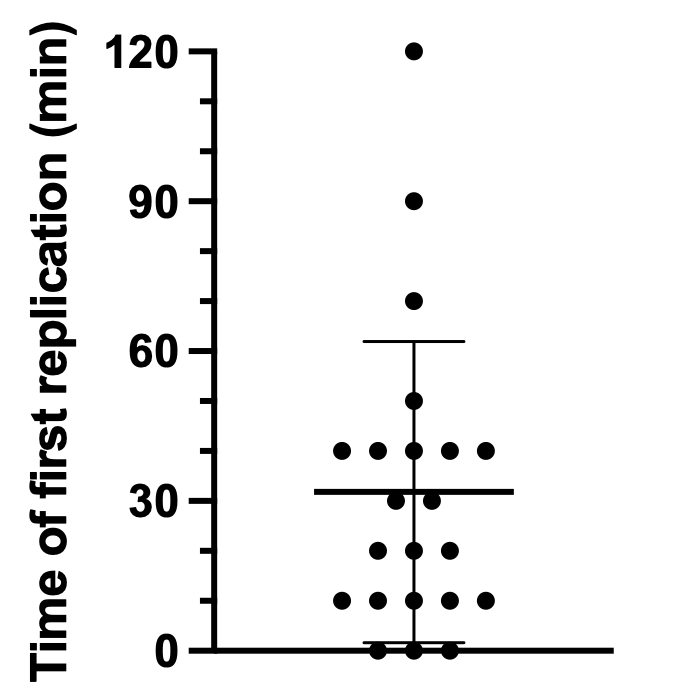

Supplement: Supplementary Figure 3 — Distribution of L. monocytogenes replication times post invasion. Shown is the time to first replication of Lm-dsRed PactA-GFP and Lm-dsRed Phly-GFP during infection of RAW 264.7 macrophages at MOI 0.25 from pooled data in . Data from 3 replicates and 22 total replicative L. monocytogenes presented as a bar graph with mean and SD. [file Image_3.tiff]

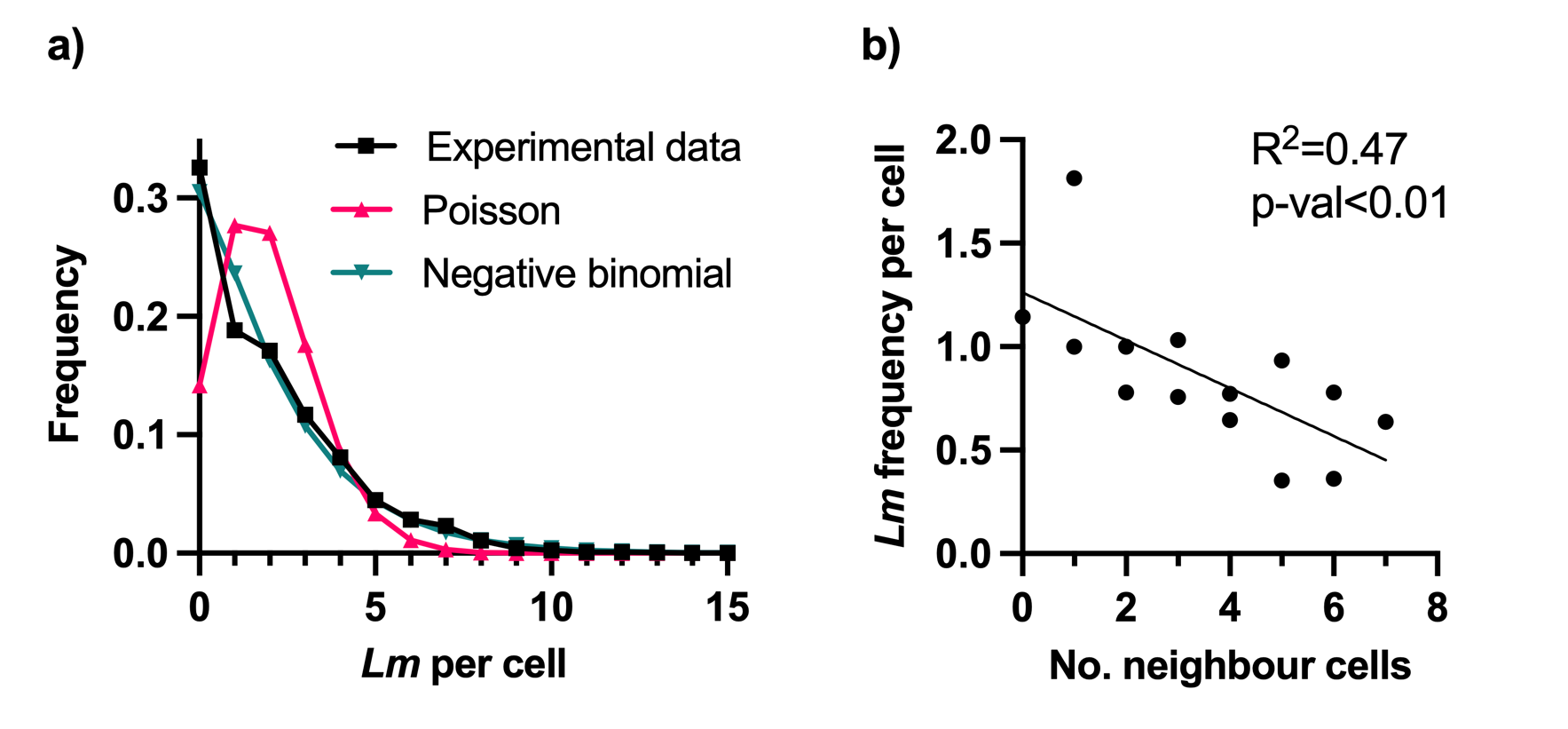

Supplement: Supplementary Figure 4 — Distribution of bacteria adhering to host cells at MOI 5. (A) Distribution of bacteria per cell at t0 when RAW 264.7 macrophages simultaneously infected with Lm-dsRed and Lm-GFP at combined MOI 5 (at 1:1 ratio). Plot shows mean frequency of distribution for experimental data (black) from 3 replicates, and expected data assuming a one parameter Poisson distribution (pink) or negative binomial distribution (teal). (B) Relationship between mean frequency of bacteria per cell at t0 and number of neighbouring cells when RAW 264.7 macrophages simultaneously infected with Lm-dsRed and Lm-GFP at combined MOI 5 (at 1:1 ratio). Data from 2 replicates (black circles) and simple linear regression (black line, R2 = 0.47, p-value = 0.007). [file Image_4.tiff]

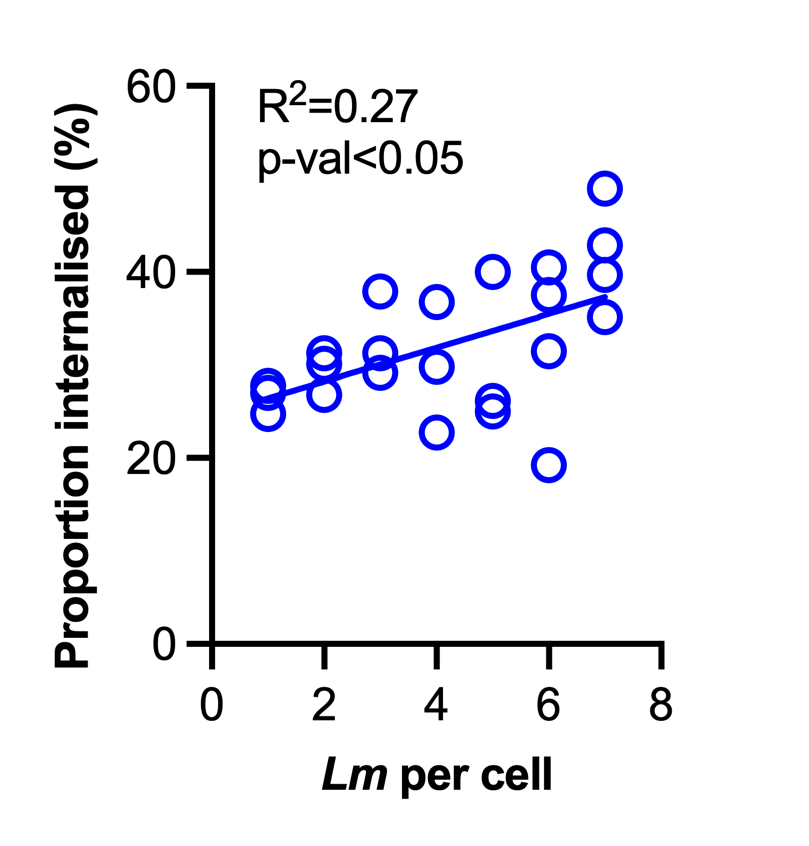

Supplement: Supplementary Figure 5 — Analysis of L. monocytogenes internalisation at MOI 5. Relationship between proportion of internalised Lm-GFP and number of Lm-GFP associated with the cell at t0 when RAW 264.7 macrophages infected at MOI 5. Individual data from four replicates (circles) and simple linear regression (solid line, R2 = 0.27, p-value = 0.01) shown. Data obtained from internalisation assay using anti-Lm 594 staining of infected cells fixed at t0 as depicted in 3b. [file Image_5.tiff]

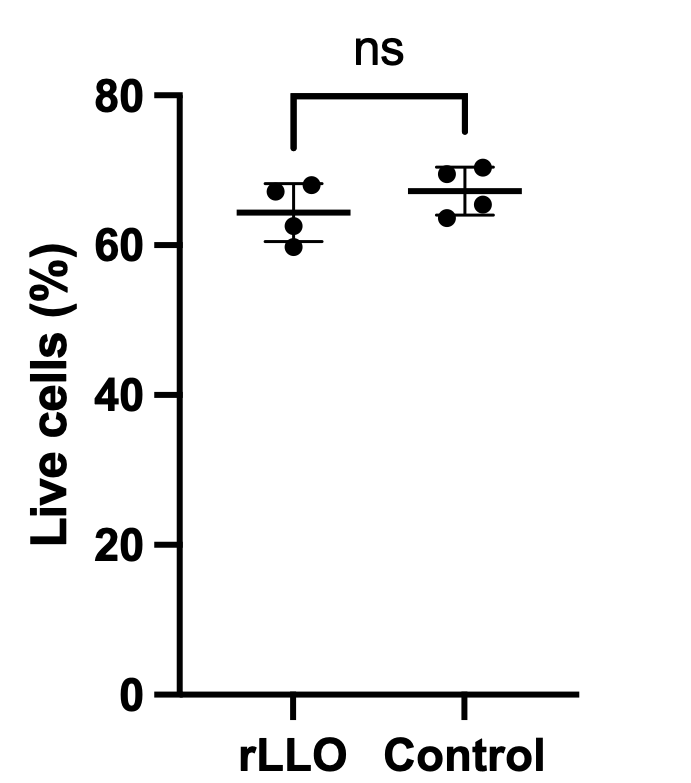

Supplement: Supplementary Figure 6 — Analysis of host cell viability upon LLO exposure. Proportion of live RAW 264.7 cell staining incubated with 0 or 2 nM rLLO. Individual data from three experiments (circles) with mean and SD (solid lines). rLLO added with inoculant and removed at t0. Statistical significance assessed with Mann-Whitney rank test (ns -not significant). [file Image_6.tiff]
